# The Monitoring of Vertical Transmission of HIV in the Northeastern Romania Cohort—A Continuing Challenge

**DOI:** 10.3390/medicina62040632

**Published:** 2026-03-26

**Authors:** Isabela Ioana Loghin, Andrei Vaţă, Șerban Alin Rusu, Ion Cecan, Otilia-Elena Frăsinariu, Victor Daniel Dorobăț, Vlad Hârtie, Carmen Mihaela Dorobăţ

**Affiliations:** 1 Department of Infectious Diseases, “Grigore T. Popa” University of Medicine and Pharmacy, 700115 Iasi, Romania; isabelabegezsan@yahoo.com (I.I.L.);; 2 Department of Infectious Diseases, “St. Parascheva” Clinical Hospital of Infectious Diseases, 700116 Iasi, Romania; 3 Emergency Clinical Hospital for Children “St. Maria”, 700309 Iasi, Romania; 4 Department of Intensive Care, National Institute for Infectious Diseases “Prof. Dr. Matei Balș”, 021105 Bucharest, Romania; 5 Department of Intensive Care, Clinical Hospital of Emergency “Prof. Dr. Nicolae Oblu”, 700309 Iasi, Romania

**Keywords:** HIV-AIDS vertical transmission, mother-to-child transmission, ART, Romanian pediatric cohort

## Abstract

*Background and Objectives.* Mother-to-child transmission (MTCT) or vertical transmission of human immunodeficiency virus (HIV) is largely preventable in settings where prevention of MTCT (PVT) strategies are consistently implemented. Romania represents a particular epidemiological context, as individuals from the historical pediatric HIV cohort have now reached reproductive age. This study assessed current PVT outcomes in northeastern Romania and explored the remaining circumstances in which transmission still occurs. *Materials and Methods.* We performed a retrospective observational analysis at the Regional HIV/AIDS Center of Iași (“Sfânta Parascheva” Clinical Hospital of Infectious Diseases), including all pregnant women living with HIV and their HIV-exposed infants followed between 2023 and 2025. Maternal data comprised age, place of residence, origin from the Romanian pediatric cohort, antiretroviral therapy (ART) adherence, and HIV RNA viral load in the third trimester. Obstetric characteristics, delivery mode, neonatal antiretroviral prophylaxis, and infant HIV RNA *PCR* results during follow-up up to 18–24 months were also evaluated. *Results.* A total of 61 HIV-positive pregnant women and 53 HIV-exposed infants were included. Viral suppression during pregnancy was documented in 59 women (96.7%), while two cases of detectable viremia in late pregnancy were linked to poor ART adherence. All women delivered by elective cesarean section, and all infants received neonatal antiretroviral prophylaxis, with Raltegravir added in selected higher-risk situations. Overall, MTCT was 3.8% (2/53). No transmission events were recorded in 2023 or 2024; both cases occurred in 2025 (15.4% of infants born that year) and exclusively in the context of maternal viremia. Women originating from the historical pediatric HIV cohort accounted for 31.1% (19/61) of pregnancies, and no transmission was observed among their infants. *Conclusions.* In northeastern Romania, PVT programs remain highly effective when maternal viral suppression is achieved. Residual transmission was confined to situations of maternal viremia driven by ART non-adherence, highlighting the continued importance of adherence support during pregnancy.

## 1. Introduction

Globally, mother-to-child transmission (MTCT) or vertical transmission of HIV remains a major public health challenge in efforts to control HIV/AIDS. With the scale-up of prevention programs, the number of children newly infected with HIV has declined dramatically in the past decade. In 2024, an estimated 120,000 children under five were newly infected with HIV worldwide, a 62% decrease from 2010 levels [1,2]. This progress reflects the impact of prevention of vertical transmission (PVT) initiatives, including widespread access to antiretroviral therapy (ART) for HIV-positive pregnant women [3,4]. By 2024, 84% of pregnant women living with HIV globally were receiving effective ART to prevent transmission to their infants. These interventions are crucial because, in the absence of prophylaxis, a pregnant person living with HIV has a 15–45% chance of transmitting the virus to her child during pregnancy, delivery, or chest feeding [5,6]. With comprehensive PVT measures, including lifelong maternal ART, safe obstetric practices (e.g., elective cesarean delivery), and infant post-exposure prophylaxis, the risk can be reduced to <1% [7]. The virtual elimination of pediatric HIV infections is therefore an achievable public health goal with adequate resources and coverage of interventions. Romania represents a distinct epidemiological setting. In the late 1980s, Romania experienced a large nosocomial HIV outbreak among infants and young children, an event often referred to as an “epidemiological accident” [7,8]. Between 1986 and 1991, approximately 10,000 children in Romania acquired HIV in hospitals and orphanages, due to practices such as transfusion of unscreened blood and the reuse of unsterilized injection equipment. This led to an unprecedented situation in which, by 1990, approximately 94% of reported AIDS cases in Romania occurred in children younger than 13 years of age, reflecting the large nosocomial pediatric HIV outbreak documented during that period [9]. Romania alone accounted for the majority of pediatric HIV cases in Europe at that time. The affected children—many of them newborns and infants at the time of infection—created a large cohort of perinatally and parenterally infected children who have now reached adulthood [10,11]. The long-term outcome of that cohort has profoundly shaped Romania’s HIV landscape [12]. Thousands of those children from the late ’80s have survived into adolescence and adulthood, benefiting from improved access to antiretroviral treatment over the years. Today, many of these survivors are entering their reproductive years and starting families [13,14]. As this cohort reaches reproductive age, prevention of mother-to-child transmission remains a national priority. Since the early 2000s, the Romanian Ministry of Health has implemented a comprehensive PVT program aligned with European guidelines, within the framework of the Romanian National Strategy for the Surveillance, Control and Prevention of HIV/AIDS Infection 2022–2030. In Romania, HIV care is delivered through a national network of nine regional HIV/AIDS reference centers, which provide specialized diagnosis, antiretroviral therapy, and long-term follow-up for people living with HIV and coordinate prevention programs, including prevention of vertical transmission [15,16,17]. Key strategies include universal HIV screening for pregnant women, full ART coverage for HIV-positive expectant mothers, scheduled cesarean deliveries to minimize peripartum transmission, strict avoidance of breastfeeding in women living with HIV according to current Romanian national prevention protocols, although several European guidelines increasingly recognize the possibility of supported breastfeeding under strict monitoring in women with sustained viral suppression and good adherence to antiretroviral therapy [18]. These interventions are provided free of charge and have dramatically reduced vertical transmission rates in the country [19]. In the 1990s, a significant portion of infants born to HIV-positive women became infected, but by 2014, the mother-to-child transmission rate in Romania had dropped to roughly 4% [20]. Local studies confirm that maternal ART, infant antiretroviral prophylaxis, and elective cesarean significantly lower the risk of vertical HIV transmission [21]. In many high-income settings, the recommended mode of delivery for women living with HIV is determined primarily by maternal viral suppression rather than HIV status alone. Current international guidelines, including those of the British HIV Association (BHIVA), the European AIDS Clinical Society (EACS), and the U.S. Perinatal HIV Guidelines, recommend planned vaginal delivery when maternal viral load is suppressed near delivery, as elective cesarean section has not shown additional benefit in preventing vertical transmission in this context and may increase surgical risks for both mother and infant. However, Romania represents a unique epidemiological context due to the large historical pediatric HIV cohort infected during the late 1980s and early 1990s. Many individuals from this cohort have now reached reproductive age and are increasingly represented among pregnant women living with HIV. Although Romanian prevention strategies are generally aligned with European guidelines, certain clinical practices—including the frequent use of elective cesarean section—reflect this particular epidemiological background and long-term management considerations. The objective of this study was to assess the outcomes of prevention of vertical transmission (PVT) strategies in pregnant women living with HIV managed at a regional HIV referral center in northeastern Romania between 2023 and 2025, and to identify maternal clinical and virological factors associated with residual vertical HIV transmission.

## 2. Materials and Methods

### 2.1. Study Design and Database Information

This retrospective observational study was conducted at the Regional HIV/AIDS Center of Iași, operating within the “Sfânta Parascheva” Clinical Hospital of Infectious Diseases. The analysis included all HIV-positive pregnant women who were registered and followed in the center, as well as their infants, during the study period from 15 January 2023 to 15 December 2025. Eligible participants included all HIV-positive pregnant women registered and followed at the Regional HIV/AIDS Center of Iași during the study period. Infant outcome analysis was performed only for mother–infant pairs with complete neonatal follow-up and virological testing available in our center.. The data were analyzed using Microsoft 365 Office Excel 2019 and Epi Info 2021.

Data were extracted from the center’s electronic medical records and paper-based registries used for routine clinical documentation, which systematically document clinical, immunological, and virological parameters for all participants under follow-up. For the mothers, the variables analyzed included maternal age at delivery, clinical stage according to CDC classification, membership in the historical Romanian pediatric cohort, plasma HIV viral load measurements during pregnancy, and the type of highly active antiretroviral therapy used throughout gestation. Obstetric characteristics were also recorded, specifically the mode of delivery (elective cesarean section versus vaginal birth). Adherence was assessed based on clinical records, pharmacy refill documentation, and missed follow-up visits, and was considered poor when treatment interruption was documented. Maternal virological status was defined using the last available HIV RNA measurement prior to delivery. In cases of missed prenatal follow-up or documented treatment interruption, no virological assessment was performed during late pregnancy; these cases were classified as unknown virological status and managed as high risk for vertical transmission according to prevention of mother-to-child transmission (PVT) clinical protocols. Infants born to women living with HIV were monitored according to the Romanian National Protocol for the Prevention of Mother-to-Child Transmission of HIV, which is implemented in accordance with international recommendations, including those of the European AIDS Clinical Society. Early infant diagnosis was performed using HIV DNA PCR testing, the preferred diagnostic method for infants younger than 18 months of age. Virological testing was performed at birth, at 1–2 months of age, and at 4–6 months of age, according to national and international guidelines. Additional follow-up testing and clinical monitoring were continued up to 18–24 months of age to definitively confirm or exclude HIV infection. Due to the small number of transmission events, multivariable regression analysis was not appropriate; therefore, associations were evaluated using Fisher’s exact test.

### 2.2. Approval for Ethics

Ethical approval was obtained from the Ethics Committee of the “St. Parascheva” Clinical Hospital of Infectious Diseases in Iasi, Romania, in January 2026 (Ethical approval no 1/10 January 2026). Informed consent forms were signed upon admission by each patient and their relatives, namely the mother. All patient data were anonymized before analysis, and no personally identifiable information was included in the study database. Data processing was conducted in accordance with institutional data protection regulations and the principles of the Declaration of Helsinki.

### 2.3. Study Setting

In the northeastern region of Romania, “Sfânta Parascheva” Clinical Hospital for Infectious Diseases from Iași treats a range of infectious diseases and has a capacity of 270 adult beds and 30 pediatric beds. This hospital also includes the HIV/AIDS Regional Center. With a capacity of 12 beds (9 for adults, 1 for a child and 1 for a newborn) in the HIV/AIDS Department, immunosuppressed participants and the newborn child of HIV-positive mothers are regularly evaluated according to EACS guidelines and protocols and CDC criteria [17].

All basic blood tests, such as hematology, biochemistry, and immunology tests, were performed in the hospital’s central laboratory, and the molecular biology lab evaluated the participants CD4+ T cell counts and HIV plasma viral loads. Plasma HIV-1 RNA was quantified using the GeneXpert^®^ platform (Cepheid 360 OS 2.1 version) to determine viral load levels. If the viral load was less than 40 copies/mL, it was declared undetectable; if it was greater than 40 copies/mL, it was considered detectable. The assay’s lower limit of detection was 40 copies/mL. Also, the newborns were evaluated for HIV antibodies using enzyme-linked immunosorbent assay (ELISA) testing.

The HIV/AIDS Regional Center in Iasi currently has 1810 participants in its active records. They are evaluated and monitored periodically to ensure adherence to antiretroviral therapy, with follow-up visits every 24 weeks or earlier, depending on the case. The medical file of the patient includes specific evaluations, HIV/AIDS stage diagnosis, ART regimens, linked disorders, CD4 T cell level, HIV viral load, imagistic investigations and blood test results.

### 2.4. Study Analysis

Statistical analysis was performed using descriptive and inferential methods appropriate for the cohort size. Continuous variables were summarized using ranges and approximate medians, while categorical variables were expressed as numbers and percentages. The primary outcome analyzed in this study was vertical HIV transmission, defined as confirmed HIV infection in infants born to women living with HIV during follow-up up to 18–24 months of age. Maternal variables evaluated in relation to infant infection status included maternal HIV clinical stage according to CDC classification, maternal viral load during pregnancy (detectable versus undetectable), and antiretroviral therapy adherence (good adherence versus documented non-adherence). Associations between categorical variables and infant HIV infection were assessed using Fisher’s exact test, which was considered appropriate given the small number of transmission events observed in the cohort. A *p*-value < 0.05 was considered statistically significant.

## 3. Results

A total of 61 HIV-positive pregnant women and 53 HIV-exposed infants were included in the analysis over the three-year study period (2023–2025) at the Regional HIV/AIDS Center in Iași, North-East Romania. All pregnant women living with HIV who presented for antenatal care during this interval were evaluated. No mother–infant pairs were excluded, and no missing data were recorded for maternal viral load monitoring, ART adherence, mode of delivery, or infant HIV RNA testing. This allowed assessment of maternal and infant outcomes. Maternal demographic and clinical characteristics are summarized in Table 1. Across all three years, maternal age fell within the expected reproductive interval. The overall median maternal age was 30 years, with a range of 24–43 years. The age distribution showed minor shifts over time: women delivering in 2023 tended to be in their late twenties to mid-thirties, while the 2024 and 2025 cohorts included a slightly younger distribution. Residential distribution remained similar across years, with 34,4% living in urban areas and 65,6% in rural regions. This cohort included women from the historical pediatric HIV cohort. In 2023, nearly half of all pregnant women (44.8%) belonged to this group, decreasing progressively in 2024 (21.0%) and 2025 (15.4%). The overall incidence proportion of mother-to-child transmission in the cohort was 3.8% (2/53 HIV-exposed infants; 95% CI: 0.5–13.0%).

Maternal virological control during pregnancy remained high throughout most of the study period. All pregnant women in 2023 and 2024 achieved undetectable HIV viral load by the third trimester, supported by documented good adherence to ART. In 2025, 11 of 13 women remained suppressed, whereas 2 had detectable viral load due to clear treatment non-adherence. These two cases represented the only maternal virological failures across the three-year period and were associated with infant infection.

Immunological staging of maternal HIV infection was available for all pregnant women. Most women were classified as CDC stage B, defined by oropharyngeal candidiasis. Specifically, 20 women were classified as stage B1, 10 as stage B2, 5 as stage A2, and 7 as stage A1, while 19 women had Acquired Immunodeficiency Syndrome (AIDS) (stage B3). All stage B classifications were determined by the presence of oropharyngeal candidiasis. Maternal HIV clinical staging recorded in the medical files followed the historical CDC 1993 classification system [22]; which was in use at the time when most participants in this cohort were initially diagnosed with HIV infection. Because many of the women had a longstanding HIV infection diagnosed prior to 2014, their clinical stage was recorded according to this earlier classification. The updated CDC 2014 surveillance case definition was taken into account when interpreting the results, noting that some conditions previously included in category B (such as oropharyngeal candidiasis) are not considered AIDS-defining illnesses under the revised definition.

Both cases of vertical transmission occurred in mothers with documented non-adherence to antiretroviral therapy and loss to routine prenatal follow-up at the Regional HIV/AIDS Center. These women did not attend the recommended periodic clinical, biological, and viro-immunological evaluations during pregnancy and did not regularly collect their antiretroviral medication from the center. At the time of delivery, maternal HIV RNA levels were 80,000 copies/mL in one case and 65,000 copies/mL in the other, confirming uncontrolled maternal viremia.

The distribution of maternal HIV clinical stages and their relationship with infant infection status are presented in Table 2. The antiretroviral single-tablet regimens administered during pregnancy are summarized in Table 3.

A significant association was observed between advanced maternal HIV clinical stage and vertical transmission. All transmission events occurred in mothers with stage B3 infection, whereas no infections were recorded among infants born to mothers in stages A1–B2 (Fisher’s exact test, *p* = 0.048).

A composite high-risk maternal profile defined by the simultaneous presence of stage B3 disease, detectable maternal viral load, and antiretroviral non-adherence perfectly identified all cases of vertical transmission. Both infected infants were born to mothers meeting all three criteria, whereas no transmission occurred in their absence (Fisher’s exact test, *p* < 0.001).

Most women included in the cohort had a previously established diagnosis of HIV infection and were already receiving long-term antiretroviral therapy prior to pregnancy, as part of routine HIV care. Continuous antenatal ART was therefore maintained throughout pregnancy in the majority of cases. In contrast, the two cases of vertical transmission occurred in mothers who had been previously diagnosed with HIV and had documented recommendations for antiretroviral therapy, but who demonstrated poor adherence and loss to clinical follow-up during pregnancy. As a consequence, they did not attend regular prenatal monitoring and had uncontrolled maternal viremia at delivery.

All deliveries were conducted via elective cesarean section, consistent with national PVT recommendations [15]. No deviations from the scheduled mode of delivery occurred. Infant characteristics and virological outcomes are presented in Table 4.

All 53 infants received neonatal antiretroviral prophylaxis immediately after birth, with specific doses of Zidovudine syrup and Lamivudine syrup (AZT/3TC) according to their weight as the standard regimen; Raltegravir (RAL) was added for higher-risk cases. All infants were formula-fed, and no breastfeeding took place. HIV RNA testing was performed using PCR-based quantitative assays available through the National AIDS Program.

In 2023 and 2024, outcomes were uniformly favorable: all infants remained HIV-negative throughout follow-up, with no transmission events recorded. In 2025, however, two infants were diagnosed with HIV infection during follow-up. Both had received complete prophylaxis and were delivered by cesarean section, but were born to a mother with a detectable viral load due to non-adherence to ART. These were the only cases of transmission observed across the entire study period. All infants born to mothers with undetectable viral load were tested HIV-negative, while transmission occurred only in the presence of detectable maternal viremia and poor adherence.

Women originating from the historical Romanian pediatric HIV cohort accounted for 31.1% (19/61) of pregnancies during the study period. This shift reflects broader demographic changes within Romania’s HIV population, as aging survivors transition out of reproductive age while adults diagnosed in the past two decades increasingly account for new pregnancies. Notably, no infants born to pediatric cohort mothers acquired HIV during the study period (Table 5).

**Table 5:** Distribution of mothers from the Romanian pediatric HIV cohort.

Year	Total Pregnant Women	Pediatric Cohort Mothers	Percentage
2023	29	13	44.8%
2024	19	4	21.0%
2025	13	2	15.4%
Total	61	19	31.1%

## 4. Discussion

This study evaluated the effectiveness of contemporary prevention of mother-to-child transmission (PVT) interventions in a regional Romanian HIV referral center and identified the determinants associated with residual vertical transmission in the modern antiretroviral therapy era [10]. The findings demonstrate that the PVT cascade was highly effective when consistently implemented; as maternal antiretroviral therapy coverage was universal, virological suppression during pregnancy was achieved in the large majority of women, and no transmission occurred among pregnancies with an undetectable maternal viral load [11]. The only transmission events observed during the study period occurred exclusively in mothers with documented treatment interruption and detectable viremia in late pregnancy, confirming maternal viral suppression as the main determinant of infant outcomes. In our study, the two cases of vertical HIV transmission occurred exclusively in mothers with documented antiretroviral therapy non-adherence and high maternal viral load at delivery. Transmission occurred despite an elective cesarean section and triple neonatal post-exposure prophylaxis. These findings reinforce the central role of maternal viral suppression as the key determinant of vertical HIV transmission risk [23]. Overall, our findings suggest that the PVT program implemented in the regional HIV center functions effectively, as reflected by high ART coverage, high rates of maternal viral suppression, universal neonatal prophylaxis, and favorable infant outcomes in the majority of cases. Importantly, the only instances of vertical transmission occurred in the context of maternal viremia and documented antiretroviral treatment non-adherence.

These results are consistent with international prevention strategies, indicating that the risk of vertical transmission decreases to below 1% when effective maternal antiretroviral therapy leads to sustained viral suppression prior to delivery [24,25]. The global expansion of antenatal HIV screening programs and universal ART availability has shifted the main challenge of PVT programs from treatment access to maintaining adherence and continuous engagement in care throughout pregnancy [26]. These long-term survivors continue to shape the demographic landscape of HIV in Romania, though their proportion among reproductive-aged women is gradually declining. Our findings support this paradigm, demonstrating that residual transmission in a well-structured prevention system occurs primarily in the context of incomplete maternal adherence.

Sibiude et al. [27] analyzed data from the French Perinatal Cohort, including more than 14,000 pregnancies among women living with HIV. The authors reported that no cases of vertical HIV transmission occurred among 5482 women receiving continuous antiretroviral therapy from conception with undetectable viral load at delivery, highlighting the effectiveness of sustained viral suppression in preventing perinatal transmission. The similarity between these national surveillance findings and our regional cohort outcomes indicates that PVT performance in northeastern Romania has reached levels comparable to those observed in Western European healthcare systems when standardized monitoring and treatment protocols are consistently implemented [28].

Recent studies have further highlighted the effectiveness of contemporary antiretroviral therapy in preventing vertical HIV transmission. Holt et al. evaluated pregnancy outcomes among women living with HIV receiving modern integrase inhibitor-based antiretroviral regimens and reported high rates of maternal viral suppression with favorable perinatal outcomes, consistent with the very low risk of vertical transmission observed in current clinical practice [29].

Other studies show that, despite the challenges related to maternal viremia and immunosuppression identified in various cases, vertical transmission rates in people living with HIV are low, ranging from 2.2% in South Africa, 1.9% in Asia, 1.6% in Spain, 1% in the USA, and 0.6% in Brazil to 0% in some European cohorts [30].

Dugdale et al. recently conducted a large systematic review and meta-analysis evaluating the relationship between maternal HIV viral load and the risk of vertical transmission. The authors demonstrated a strong dose–response relationship between maternal viraemia and transmission risk, with transmission rates of approximately 0.2% when maternal viral load was <50 copies/mL, increasing to 1.3% at viral loads between 50 and 999 copies/mL, and reaching around 5% when viral load exceeded 1000 copies/mL. Importantly, no cases of vertical transmission were reported among women who initiated antiretroviral therapy before pregnancy and maintained sustained viral suppression until delivery. In our study, similar findings were observed. All cases of mother-to-child HIV transmission occurred exclusively in mothers with detectable viraemia at delivery, with HIV RNA levels of 80,000 copies/mL and 65,000 copies/mL. Both cases were associated with documented non-adherence to antiretroviral therapy and absence from regular clinical monitoring during pregnancy. Conversely, no cases of transmission occurred among women with sustained viral suppression, supporting the well-established role of maternal viral load as the primary determinant of vertical HIV transmission risk [31].

Neonatal antiretroviral prophylaxis was uniformly administered to all infants in our cohort; however, the occurrence of infection in cases of maternal viremia indicates a principle consistently emphasized in international PVT research: neonatal prophylaxis alone cannot compensate for uncontrolled maternal infection [28]. Instead, infant prophylaxis functions as an additional protective measure complementing maternal viral suppression, rather than replacing it [26]. These observations highlight the importance of strengthening adherence-support strategies during pregnancy, particularly in participants with previous treatment interruptions or inconsistent healthcare engagement.

The demographic structure of the cohort also provides insight into the evolving epidemiology of HIV in Romania. A proportion of pregnancies occurred in women originating from the historical Romanian pediatric HIV cohort, although their representation declined progressively during the study period, reflecting the demographic transition of the national epidemic. Favorable pregnancy outcomes observed in these women, particularly the absence of transmission when virological suppression was achieved, demonstrate that long-term survivors with complex treatment histories can successfully complete pregnancy without transmission risk when retained in specialized care programs with continuous virological monitoring. Pregnancies among women with perinatally acquired HIV infection represent an increasingly recognized clinical scenario as long-term survivors of childhood HIV infection reach reproductive age. Foster et al. reported that individuals living with lifelong HIV infection often present complex antiretroviral treatment histories and require careful monitoring during pregnancy due to potential challenges related to long-term immunological status and treatment adherence. In our cohort, women originating from the historical Romanian pediatric HIV cohort represented 31.1% of all pregnancies. Importantly, none of the infants born to these women acquired HIV infection during the study period, suggesting that when sustained viral suppression and specialized multidisciplinary care are maintained, favorable pregnancy outcomes can be achieved even among women with lifelong HIV infection [30].

Fleșeriu et al. reviewed the current evidence regarding maternal HIV infection and antiretroviral therapy during pregnancy and emphasized that the widespread use of antiretroviral therapy has dramatically reduced the risk of mother-to-child transmission, with transmission rates falling to below 1% in many settings when maternal viral suppression is achieved [32]. In our cohort, similar findings were observed. No cases of vertical HIV transmission occurred among women with sustained viral suppression during pregnancy, whereas the two transmission events occurred exclusively in mothers with detectable viraemia at delivery (80,000 and 65,000 copies/mL) in the context of documented antiretroviral therapy non-adherence. These observations further support the central role of maternal viral load control as the key determinant of vertical HIV transmission risk.

Several limitations should be acknowledged. The relatively small number of pregnancies reflects the epidemiological profile of a regional HIV referral center and means that individual transmission events may have a greater proportional impact on the calculated transmission rate. In addition, the retrospective design limited the detailed assessment of psychosocial factors influencing treatment adherence, which may have contributed to the observed cases of virological failure. Nevertheless, the study benefits from complete follow-up of all mother–infant pairs, standardized laboratory monitoring, and the absence of missing outcome data, providing a reliable assessment of prevention of vertical transmission outcomes within the regional healthcare system.

The absence of late-pregnancy virological measurements in these cases reflects loss to follow-up rather than missing study data and represents a recognized clinical risk factor for vertical transmission in PVT cohorts. Overall, our findings indicate that the PVT program in northeastern Romania is highly effective when maternal ART adherence and viral suppression are maintained. In agreement with international cohort studies, vertical transmission in the contemporary ART era is now primarily associated with lapses in maternal treatment adherence rather than limitations of available preventive interventions. Strengthening adherence-support programs, ensuring early antenatal engagement in care and maintaining multidisciplinary follow-up of pregnant women living with HIV should therefore represent the main priorities for sustaining zero-transmission outcomes and advancing toward the long-term objective of eliminating pediatric HIV infection.

## 5. Conclusions

This study suggests that the prevention of mother-to-child transmission of HIV in Northeast Romania is effective when comprehensive PVT protocols are consistently applied and maternal adherence to ART is maintained. Across the three-year observation period, universal ART coverage, sustained maternal virological suppression, elective cesarean delivery, and complete neonatal prophylaxis resulted in excellent outcomes, with no HIV transmission in 2023 and 2024 and only two transmission events in 2025, both occurring exclusively in the context of documented maternal non-adherence. These findings confirm that, in the current Romanian healthcare setting, vertical HIV transmission has become a rare preventable event, and they reinforce the central role of maternal viral load control as the key determinant of infant outcome. Continued strengthening of adherence support, early engagement in care, and sustained long-term follow-up of women living with HIV—including those originating from the historical Romanian pediatric cohort—remain necessary to further advance Romania toward elimination of pediatric HIV infection.

## Figures and Tables

**Table 1:** Maternal demographic and clinical characteristics, 2023–2025.

Variable	2023 (*n* = 29)	2024 (*n* = 19)	2025 (*n* = 13)	Total (*N* = 61)
Age, median (IQR), years	31 (25–43)	29 (24–35)	28 (25–30)	30 (24–43)
Urban residence, *n* (%)	10 (34.5)	7 (36.8)	4 (30.8)	21 (34.4)
Rural residence, *n* (%)	19 (65.5)	12 (63.2)	9 (69.2)	40 (65.6)
Pediatric cohort mothers, *n* (%)	13 (44.8)	4 (21.0)	2 (15.4)	19 (31.1)
Adult-acquired HIV, *n* (%)	16 (55.2)	15 (79.0)	11 (84.6)	42 (68.9)
ART adherence—Yes, *n* (%)	29 (100)	19 (100)	11 (84.6)	59 (96.7)
ART adherence—No, *n* (%)	0 (0)	0 (0)	2 (15.4)	2 (3.3)
Undetectable viral load, *n* (%)	29 (100)	19 (100)	11 (84.6)	59 (96.7)
Detectable viral load, *n* (%)	0 (0)	0 (0)	2 (15.4)	2 (3.3)
Delivery mode—Cesarean section, *n* (%)	29 (100)	19 (100)	13 (100)	61 (100)
Duration of HIV infection < 5 years, *n* (%)	(0) 0	10 (52.6)	(0) 0	10 (16.4)
Duration 6–9 years, *n* (%)	16 (55.2)	5 (26.3)	11 (86.4)	32 (52.5)
Duration ≥10 years, *n* (%)	13 (44.8)	4 (21.1)	2 (15.4)	19 (31.1)

**Table 2:** Maternal HIV clinical stage and vertical transmission.

Maternal HIV Clinical Stage	Number of Mothers (*n* = 61)	Percentage (%)	HIV-Positive Infants (*n* = 2)
A1	7	11.5	0
A2	5	8.2	0
B1	20	32.8	0
B2	10	16.4	0
B3 (AIDS)	19	31.1	2
Total	61	100	2

**Table 3:** Antiretroviral single-tablet regimens during pregnancy.

Antiretroviral Regimen (Active Compounds)	Number of Mothers (*n* = 61)	Percentage (%)
Bictegravir + emtricitabine + tenofovir alafenamide	25	41.0
Doravirine + lamivudine + tenofovir disoproxil fumarate	17	27.9
Dolutegravir + lamivudine	19	31.1
Total	61	100

**Table 4:** Infant characteristics and virological outcomes, 2023–2025.

Variable	2023 (*n* = 21)	2024 (*n* = 19)	2025 (*n* = 13)	Total (*n* = 53)
Neonatal prophylaxis	100%	100%	100%	100%
Prophylaxis regimen	AZT+3TC; RAL high-risk	AZT+3TC	AZT+3TC; RAL	—
HIV RNA first test	All negative	All negative	11 neg; 2 pos	51 neg; 2 pos
HIV RNA at 18–24 months	21 negative	19 negative	11 negative	51 negative
Confirmed HIV infections	0	0	2	2
Transmission rate	0%	0%	15.4%	3.8%

## Data Availability

The datasets presented in this article are not readily available because the data are part of an ongoing study or due to technical limitations. Requests to access the datasets should be directed to contact corresponding author.

## Abbreviations

The following abbreviations are used in this manuscript:

MTCT	Mother-To-Child Transmission
PVT	Prevention Of Mother-To-Child Transmission
HIV	Human Immunodeficiency Virus
AIDS	Acquired Immunodeficiency Syndrome
